# Overview of Systematic Reviews: Yoga as a Therapeutic Intervention for Adults with Acute and Chronic Health Conditions

**DOI:** 10.1155/2013/945895

**Published:** 2013-05-16

**Authors:** Marcy C. McCall, Alison Ward, Nia W. Roberts, Carl Heneghan

**Affiliations:** ^1^Department of Continuing Education, Kellogg College, University of Oxford, 60-62 Banbury Road, Oxford OX2 6PN, UK; ^2^Department of Primary Care Health Sciences, University of Oxford, New Radcliffe House, Walton Street, Jericho OX2 6NW, UK; ^3^Bodleian Health Care Libraries, University of Oxford, Old Road, Headington OX3 7LE, UK

## Abstract

*Objectives*. Overview the quality, direction, and characteristics of yoga interventions for treatment of acute and chronic health conditions in adult populations. *Methods*. We searched for systematic reviews in 10 online databases, bibliographic references, and hand-searches in yoga-related journals. Included reviews satisfy Oxman criteria and specify yoga as a primary intervention in one or more randomized controlled trials for treatment in adults. The AMSTAR tool and GRADE approach evaluated the methodological quality of reviews and quality of evidence. *Results*. We identified 2202 titles, of which 41 full-text articles were assessed for eligibility and 26 systematic reviews satisfied inclusion criteria. Thirteen systematic reviews include quantitative data and six papers include meta-analysis. The quality of evidence is generally low. Sixteen different types of health conditions are included. Eleven reviews show tendency towards positive effects of yoga intervention, 15 reviews report unclear results, and no, reviews report adverse effects of yoga. Yoga appears most effective for reducing symptoms in anxiety, depression, and pain. *Conclusion*. Although the quality of systematic reviews is high, the quality of supporting evidence is low. Significant heterogeneity and variability in reporting interventions by type of yoga, settings, and population characteristics limit the generalizability of results.

## 1. Introduction

Over 30 million people practice yoga, a spiritual and health discipline of Indian origin [1]. In January 2007, *yoga therapy* was defined as the “process of empowering individuals to progress toward improved health and well-being through the application of the philosophy and practice of Yoga” [2]. Nearly 14 million Americans (6.1% of the population) say that a doctor or therapist has recommended yoga to them for their health condition [3]. In the United Kingdom, national healthcare services promote yoga as a safe and effective way to promote physical activity, improving strength, balance, and flexibility as well as a potential benefit for people with high blood pressure, heart disease, aches and pains, depression, and stress [4]. 

 Yoga research in medical health literature continues to increase. Over 2000 journal articles in yoga therapy have been published online (http://www.ncbi.nlm.nih.gov/pubmed). In 2012, 274 new yoga articles were added to PubMed, with 46 results after a “systematic review” title search on the US National Library of Medicine. However, the quality and direction of evidence for yoga therapy is unclear. In one clinical review, results show psychological symptoms and disorders (anxiety, depression, and sleep), pain syndromes, autoimmune conditions (asthma, diabetes, and multiple sclerosis), immune conditions (lymphoma and breast cancer), pregnancy conditions, and weight loss are all positively affected by yoga [6]. An overview from 2010 includes 21 systematic reviews that yield unanimous positive results for just two conditions—cardiovascular risk reduction and depression [7].

 The aim of this overview is to systematically collect, summarize, and evaluate key findings in yoga systematic reviews to determine the strength of evidence in adult health conditions. Components of yoga interventions, the quality and direction of evidence will be investigated for the first time. 

## 2. Methods

### 2.1. Criteria for Considering Reviews for Inclusion

#### 2.1.1. Types of Reviews

Systematic reviews of yoga as a primary intervention to treat any health condition with at least one randomized-controlled trial (RCT) of yoga are included. Any review assessing multiple health conditions is excluded. Included reviews must satisfy all Oxman criteria as follows: state a replicable search method; adequately attempt to retrieve all relevant data; collect the data in a systematic way; analyze and present the results appropriately; consider sources of bias and the quality of evidence [48]. To allow for sufficient in-depth analysis of each systematic review, publications after June 1, 2012, are not included though considered in the discussion and limitations of the overview. 

#### 2.1.2. Types of Participants

As the population of interest, adult participants with a diagnosed and existing acute or chronic health condition are included. Systematic reviews with asymptomatic or otherwise healthy participants and children (<18 years) are excluded to limit the heterogeneity in an already comprehensive overview.

#### 2.1.3. Types of Interventions

Any type of yoga as defined by review authors compared to a control group receiving no intervention or interventions other than yoga is included. A definition for *yoga* or *yoga therapy* in research has not been standardized though for the purposes of this overview, authors define yoga as “any movement meditation technique that includes breathing techniques (pranayama) or one or more of the following: physical postures specific to yoga, meditation or chanting (mantra) in the name of yoga.” Allied health or healing arts that are similar to, but do not call themselves, yoga are not included. Martial arts or alternative healing modalities including Karate, Tai Chi, Qigong, reiki, massage, stretching alone, pilates, and acupuncture are not included. Talk therapies including psychological, social, and cognitive behavioral modification strategies are excluded. Systematic reviews that include multiple interventions with yoga are included when the yoga data can be isolated. 

### 2.2. Outcomes

After consultation amongst the authors (M. C. McCall, C. Heneghan, A. Ward), the following list of outcomes are identified for analysis and will be included if authors note them as either primary or secondary outcomes.

#### 2.2.1. Primary Outcomes

- All-cause mortality.
- Direction and magnitude of disease progression.
- Surrogate markers and biomarkers that correlate with disease progression (i.e., blood pressure, resting heart rate, and endocrine levels).
- Number of clinical visits and/or hospital utilization rates.
- Changes in medication or prescription patterns.

#### 2.2.2. Secondary Outcomes

- Self-reported measures of health, coping or other (i.e., HRQL).
- Psychosocial or behavioral outcomes.
- Cost effectiveness and related evaluations.

### 2.3. Search Methods for Identification of Reviews

An electronic search of 10 online health databases including Medline, Cochrane Library, and CINAHL was designed by combining natural language and MeSH terms for yoga as the key components, see the Appendix (M. C. McCall, N. Roberts). In addition, hand-searches of relevant journals and journalistic books including The Science of Yoga [49] and Yoga as Medicine [50] were conducted. Websites of known yoga research institutes were visited. References and bibliographies of found reviews were searched for additional titles. 

### 2.4. Data Collection and Analysis

#### 2.4.1. Selection of Reviews

 The first reviewer screened titles, abstracts, and full articles found from electronic and other sources. A second reviewer (C. Heneghan) provided supervision and random assessment of the selection process. 

#### 2.4.2. Data Extraction and Management

One reviewer (M. C. McCall) systematically collected and extracted the data to standardized digital collection forms. Two other reviewers (C. Heneghan, A. Ward) independently assessed the accuracy of the data collection. Consensus through discussion or eventual consultation of a third-party resolved any discrepancies. Any missing data is considered a limitation of the overview. In reviews that include multiple interventions and yoga, data is collected on a separate database to allow for independent analysis. In multiple intervention reviews, only yoga-specific data is reported. 

### 2.5. Assessment of Methodological Quality of Included Reviews

We address two aspects of quality for the included reviews: the quality of evidence included in the reviews and the quality of the systematic reviews themselves. The first reviewer performed the quality assessments with supervision from a second author. 

#### 2.5.1. Quality of Evidence in Included Reviews

The authors sought to record “Grade of Recommendations Assessment, Development and Evaluation” (GRADE) from systematic reviews. When other measures of quality were employed, judgments by first author (M. C. McCall) were made to downgrade or upgrade the quality of evidence based on the amount of potential bias due to study design and other criteria specified in the GRADE toolbox [51]. Insufficient data was reported in instances where adequate information was unavailable.

#### 2.5.2. Quality of Included Reviews

The authors implemented the “assessment of multiple systematic reviews” (AMSTAR) measurement tool [52].

### 2.6. Data Synthesis

Characteristics of all included reviews and the overview of reviews tables summarize the key findings of data collection. The summary of results includes a narrative analysis and quantitative information, where possible. Given sufficient data, the following subgroups are identified for analysis: gender, age, ethnicity, interventions by type of practice, mode of delivery, setting, duration of sessions, duration of interventions, and intensity in terms of physiological effort such as caloric expenditure or cardiovascular output.

## 3. Results

### 3.1. Description of Included Reviews

**Figure 1:**
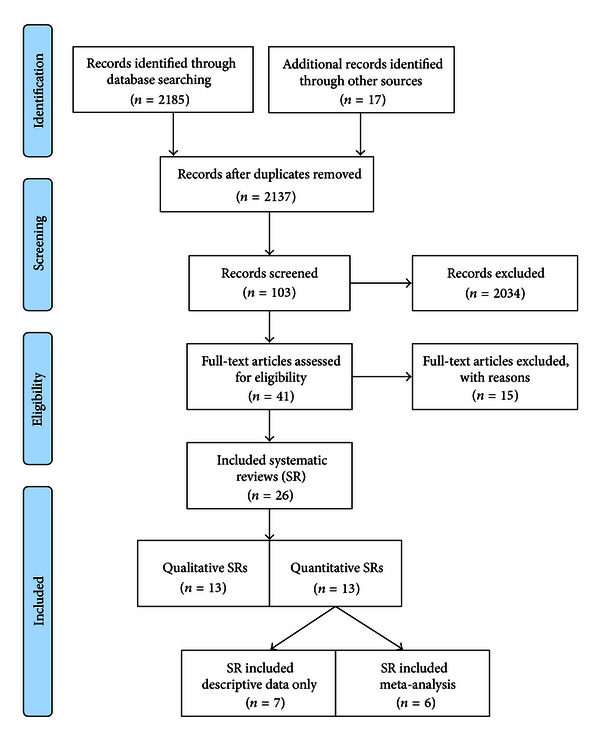
Flowchart of systematic review selection [5].

Twenty-six systematic reviews are included in this overview. Six systematic reviews provide quantitative data with meta-analyses, seven reviews provide descriptive data with no pooled analysis, and 13 reviews contain qualitative descriptions of results. Twelve systematic reviews include only yoga interventions.  Figure 1 outlines the selection process in an article flow diagram. Refer to Table 1 for characteristics of included reviews. See additional Table 2 for full list of reviews and reasons for exclusion. The systematic reviews include evidence from 125 primary studies, of which 92 studies include only yoga interventions. 

#### 3.1.1. Population

The total number of participants across all studies is 5915. Six reviews do not include studies with sample sizes greater than 50 participants at baseline. The age range of participants is 18 to 77 years. Mean age, gender, ethnicity, or socioeconomic status of the sample population is unavailable due to insufficient reporting, although the majority of participants are women. 

Twelve systematic reviews investigate only yoga interventions and include the following health conditions: anxiety (4 reviews), pain management (2 reviews), with one review each in depression, epilepsy, psychiatric disorder, diabetes, arthritis, and relief of menopause symptoms. The 14 systematic reviews that include yoga therapy in combination with other interventions measured health outcomes in carpal tunnel syndrome and diabetes risk factors (2 reviews each), with one review each in anxiety, asthma, chronic kidney disease, fibromyalgia, hypertension, low back pain, menopause, pain management in labor, chronic pain, and osteoarthritis.

#### 3.1.2. Length of Intervention and Followup

Of 25 reporting systematic reviews, one (with 2 primary studies) includes only trials ≥24 weeks duration. Follow-up measures are mentioned in eight of the 26 reviews, where four report on primary studies that include follow-up measures ≥12 weeks, two report follow-up measures <12 weeks, and two report no follow-up evaluations.

#### 3.1.3. Characteristics of Intervention

Twenty-two systematic reviews include any type of yoga intervention. Two systematic reviews include only Kundalini yoga [18, 19] one systematic review each includes only Restorative yoga [9] and Yoga of Awareness [20]. The other types of yoga intervention are listed in Box 2 include: Viniyoga, Integrated yoga, Raj, Iyengar, Kriya, Sahaja, Siddha Samadhi, hot, water, and Tibetan yoga. Modified, non-descriptive, or unspecified yoga interventions are included in 12 systematic reviews. Interventions of Ashtanga, power, or flow yoga are not found. The most prevalent yoga intervention by type includes Iyengar (9 reviews), Hatha (7 reviews) Restorative (5), and Kundalini and Integrated yoga (3 reviews each). 

Nine of the systematic reviews do not report on the type of delivery mechanism of yoga used in their primary studies. Instructor-led yoga is identified in a majority of cases (17 reviews), independent or home study (13 reviews), book-led yoga (5 reviews), audio-led yoga (4 reviews), and video-led yoga in one review. No review evaluates the effect of yoga by type or delivery mechanism for a specific health condition. Twenty reviews report the duration and frequency of yoga sessions. The duration of yoga sessions varies between 20 and 300 minutes, an intervention of 60 minutes in length most prevalent. Seven reviews include yoga interventions with <3 yoga sessions per week, three reviews include only yoga interventions with ≥3 sessions per week, and 10 reviews include both frequencies of yoga sessions. Systematic reviews do not report on the intensity of yoga interventions in terms of physiological effort such as cardiac output or caloric expenditure.

#### 3.1.4. Comparisons

Fourteen of the 26 systematic reviews (28 primary studies) report a waitlist as comparison for treatment for yoga. Other kinds of exercise are compared to yoga in 11 systematic reviews (19 primary studies), nine systematic reviews (16 primary studies) identify usual care, while medicinal intervention is noted in three reviews (4 primary studies). Four systematic reviews (19 studies) do not report the use of control groups or comparisons. Other comparisons reported in the reviews include disseminating reading material (5 reviews, 5 studies), sham yoga (3 reviews, 5 studies), talk therapy (2 reviews, 3 studies), and lectures (2 reviews, 2 studies). 

### 3.2. Methodological Quality of Included Reviews

#### 3.2.1. Quality of Included Reviews

The overall quality of systematic reviews is high (AMSTAR average = 9.4). Fifteen of the reviews are considered of very high quality (AMSTAR ≥ 10), 6 of high quality (AMSTAR 8–9.9), 5 reviews of medium quality (4–7.9 AMSTAR), and no systematic review scores below 4 points. See Table 3 for the AMSTAR ratings of the included systematic reviews. All 26 reviews scored in five of eleven methodological criteria including (refer to Box 1): identification of a priori design, using duplicate referees for study selection and data extraction, implementing a comprehensive literature search, considering the status of publication for inclusion, and the assessment and documentation of the scientific quality of evidence. The characteristics of included studies, respective quality, and the methods to combine findings of those studies are appropriate in 21 reviews. Lists of excluded studies and conflicts of interest are inconsistently reported (16 reviews only). A statistical investigation to determine a likelihood of bias is most poorly reported (2 of 12 yoga—only reviews). 

#### 3.2.2. Quality of Evidence in Included Reviews

The quality of evidence ranges from very poor/low to moderate quality (see Table 3). No high-quality evidence is included in the reviews. Systematic review authors implement a diverse set of tools to evaluate evidence, including Jadad scores, CONSORT guidelines, and PEDro scales. In 16 systematic reviews, the GRADE approach is applied to uniform results, while 10 reviews did not provide sufficient data to independently assess their quality of evidence. 

### 3.3. Effects of Interventions

#### 3.3.1. All-Cause Mortality

Outcome results for all-cause mortality are not studied in the reviews. The absence of data could be due to characteristics of study design including length of trials (typically 3–6 months) and small sample sizes (*n* < 50). The population samples usually include middle-aged adults receiving treatment for chronic illnesses; thus, mortality may be limited in such groups, or yoga therapy may have no effect on reducing mortality. 

#### 3.3.2. Direction and Magnitude of Disease Progression

Nine reviews measure the direction and magnitude of disease progression. These chronic diseases include anxiety [18, 19], depression [27], treatment of psychiatric disorder [11], clinical outcomes in arthritis [14] and osteoarthritis [23], carpal tunnel syndrome [26], epilepsy [30], and asthma [29]. Included studies of yoga therapy are characteristically short in duration, which will contribute to the lack of available evidence to analyze this outcome. 

#### 3.3.3. Surrogate Markers and Biomarkers That Correlate with Disease Progression (i.e., Blood Pressure, Resting Heart Rate, and Endocrine Levels)

Five systematic reviews measure surrogate markers that correlate with disease progression including blood pressure [12], body mass index [9], metabolic and anthropometric measures for diabetes mellitus [16], fasting blood glucose [8] and muscular strength [15]. Higher quality research with controlled clinical trials report a 6.9% reduction in fasting glucose of adults with diabetes and 7.8% reduction in body weight, with reductions in systolic and diastolic blood pressures ranging from 3.9 to 13.9% and 5.8 to 15.8% for adults with diabetes or at risk of CVD [16]. Although an average decrease of 3/5 mmHg is found in hypertensive patients, Dickinson et al. suggest no good evidence exists to confirm yoga therapy is effective for treatment of hypertension as studies are too small to detect any effect on morbidity or mortality. Study designs lack blinding and use inadequate randomization techniques, thus potential biases and limitations characterizing most of these studies hinder interpretation of findings [8, 9, 15, 16].

#### 3.3.4. Number of Clinical Visits and/or Hospital Utilization Rates

Systematic reviews do not report changes in number of clinical visits and/or hospital utilization rates with yoga intervention. Although a number of interventions are implemented in a clinical setting (9 of 26 reviews), it is possible that primary researchers did not collect data regarding hospital referral rates, perhaps due to limited resources or short-time horizons. 

#### 3.3.5. Changes in Medication or Prescription Patterns

Two systematic reviews measure changes in medication with yoga intervention [16, 28]. One author concludes that yoga may be beneficial in decreasing medication usage in diabetes [16]; the second study concludes with caution that yoga may decrease medication usage in pain conditions, although results were not statistically significant [28]. 

#### 3.3.6. Self-Reported Measures of Health, Coping or Other (i.e., HRQL)

Twelve systematic reviews include self-reported measures for pain management [10, 13, 20, 22, 24, 25, 28, 31, 33], menopausal symptoms [17, 21], perceived stress [25], psychological wellbeing, and quality of life for cancer patients [22, 32]. Seven review authors conclude positive effects [10, 17, 20, 22, 24, 28, 32]. One RCT with treatment of low-back pain shows that Iyengar yoga (*n* = 60) can reduce pain intensity (64%), functional disability (77%), and pain medication usage (88%) versus the education control group with usual care [10]. The overview of various pain conditions (headaches, back pain, muscle soreness, labor, and arthritis) yields a moderate effect size of yoga as measured by visual analog scales and questionnaires (VAS, CMDQ, and PPI) at SMD −0.74 (95%CI, − 0.97  to − 0.52; *P* < 0.0001) [10]. Quality of life for cancer patients in yoga groups approaches significance (*P* = 0.06) with an SMD −0.29 (95% CI, −0.58 to 0.01) while psychological health outcomes (anxiety, depression, distress, stress) show a pooled effect size of SMD −0.95 (95%  CI, − 1.63  to − 0.27; *P* = 0.006) as measured by HADS, PSS, STAI, POMS, CES-D, PANAS, IES, SCL-90-R, SOSI and the distressed mood index. An earlier review (search date of April 2008) reports encouraging preliminary results for cancer patients with effect sizes that range from 0.04 to 4.67 (anxiety) and 0.17 to 7.44 (depression) in favor of yoga with concurrent treatment, though statistical significance and measuring tools are not reported [32].

Attributed to the lack of scientific rigor in large-scale and long-term studies, four reviews conclude neutral or unknown effects of yoga intervention for pain in carpal tunnel syndrome [13], pain in low back [31], in older adults [25], and for labor management [33]. 

#### 3.3.7. Psychosocial or Behavioral Outcomes

Systematic reviews do not report results on psychosocial or behavioral outcomes. 

#### 3.3.8. Cost Effectiveness and Related Evaluations

Systematic reviews do not include results on cost effectiveness and related evaluations. This narrow focus is in part due to early research development and potential lack of funding to implement trials with several outcome measures.

### 3.4. Quantitative Reports

#### 3.4.1. Meta-Analyses

Of the six reviews that included a meta-analysis of results, three investigate outcomes in pain [10, 20, 31], one review each in psychiatric disorders [11], menopausal symptoms [21], and psychological health in cancer patients [22]. For pain studies, interventions include Hatha, Iyengar, Yoga of Awareness, water yoga, Viniyoga, and unspecified yoga programs. Comparisons with physical activity, education sessions, waiting lists, routine care, and talk therapy show unanimously positive results for yoga in pain reduction [10, 20, 31]. These results suggest a moderate effect size of yoga to reduce acute pain in adult populations SMD −0.74 (95% CI, −0.97 to −0.52), in fibromyalgia patients SMD −0.54 (95% CI, −0.96 to −0.11) and low-back pain versus education, self-care, and no exercise. Conversely, yoga did not indicate positive results for menopausal symptoms including pain, psychological wellbeing, and quality of life [21].

As an adjunct therapy, Cabral et al. conclude that yoga improves treatment of depression, anxiety, posttraumatic stress disorder (PTSD), and schizophrenia, with a pooled effect size of SMD −3.25 (95%  CI, − 5.36  to − 1.14; *P* = 0.002). Pranayama techniques are implicated as most important for anxiety and stress-related disorders [11]. See Table 4 for overview of reviews with pooled results.

#### 3.4.2. Independent Study Reports (No Pooled Analysis)

Descriptive quantitative data of yoga primary studies is provided in seven reviews. Three of these reviews test the direction and magnitude of disease progression with yoga intervention for anxiety [18], asthma symptoms [29], and seizure frequency in epileptics [30]. Heiwe and Jacobson [15] measure muscular strength for chronic kidney disease patients. Self-reported measure of pain is included in two reviews [13, 32] and perceived stress [24]. 

Anxiety outcome measures in the quantitative reviews include Y-BOCS, HAS, IPAT, TAS, ACL, STAI, and SNAQ (see Box 3). In general, review results show small reduction in means for yoga groups versus control groups, although the study design varies. One nonrandomized controlled study (*n* = 71) reports anxiety neurosis (HAS) decreases with yoga treatment versus placebo capsule SMD 0.89 (95%  CI, 0.34  to  1.44; *P* = 0.001). A smaller randomized control trial measures Y-BOCS (*n* = 22) reports SMD 1.10 (95%  CI, − 0.02  to  2.22; *P* = n.r). In patients with cancer, a number of yoga interventions decrease anxiety scores (HADS, PSS, STAI SOSI, POMS, and SCL-90-R). One study reports a decrease of anxiety of SMD −0.76 (95%  CI, − 1.34  to − 0.19; *P* = 0.009) in comparison to wait-list controls. In the two reviews that assess clinical anxiety as an outcome (*n* = 1087), results range from having no beneficial effect on STAI scores SMD 0.33 (95% CI, −0.31 to 0.97) to a significant effect size of SMD −4.78 (95%  CI, − 5.83  to − 3.74; *P* = n.r) on HADS and PSS validated questionnaires. Variations in scientific characteristics including the type and duration of intervention and size of samples may account for the variation in results. Weekly Tibetan yoga showed no benefit, while integrated yoga methods including asana, pranayama, and guided relaxation for 90 minutes per week show the greatest benefit in anxious participants.

In pain reviews, Gerritsen et al. review conservative treatment outcomes for carpal tunnel syndrome and report no significant differences in pain after 8 weeks of yoga intervention. Smith et al. [33] suggest that women receiving yoga report increased satisfaction with pain relief, increased satisfaction with the childbirth experience with reduced pain intensity outcomes in self-reported visual analogue scales (VASTC, MCQ, VASPS) of MD −6.12 (95%  CI, −11.77  to − 0.47; *P* = 0.034) in latent phase labor versus usual care (*n* = 66). See Box 4 for summary of measures for pain outcomes.

In asthmatic populations, one small study (*n* = 36) reports a decrease in exacerbations (episodes per week) WMD −1.27 (95% CI, −2.26 to 0.28) following yoga breathing techniques, although results are not statistically significant [29]. The hypothesis that yoga breathing can reduce asthmatic episodes is neither confirmed nor refuted with results and further randomized controlled trials are requested. 

In one study of epileptic patients (*n* = 20), sahaja yoga intervention (versus sham yoga) increases probability of being seizure-free following six months of treatment by 40% with OR 14.54 (95%  CI, 0.67  to  316.69; *P* = 0.089). The same study shows a greater than 50% reduction of seizure duration after six months in 7 of 10 yoga participants versus 0 of 10 sham yoga participants, OR 45.00 (95%  CI, 2.01  to  1006.75; *P* = 0.016). The review author includes a second study that compares Acceptance Commitment Therapy (ACT) and yoga in-seizure outcomes. Five of 10 ACT participants versus 4 of 8 yoga participants are seizure-free after six months, with 50% or greater reduction in seizure duration in 6 of 10 (ACT) and 4 of 8 (yoga) groups, respectively. The review authors conclude that no reliable conclusions can be drawn regarding the efficacy of yoga for treatment of epilepsy due to the small number and size of studies. 

In a review on chronic kidney disease populations, a small yoga study (*n* = 37) does not show any significant increase in muscular strength for yoga versus control (no exercise/placebo exercise). This review studies a special population in which yoga-related studies are limited.

### 3.5. Subgroup Analysis

The most commonly cited health outcomes in yoga research are self-reported measures in pain (7 reviews), anxiety (6 reviews), and diabetes management (3 reviews). Five reviews measuring pain outcomes after yoga intervention report positive results. Iyengar (9 reviews), Hatha (7 reviews), and Restorative yoga (5 reviews) through instructor-led sessions (17 reviews) are most common in yoga interventions by type. Six positive effects are concluded in each of the groups of Hatha and Iyengar systematic reviews. 

The Büssing et al. review includes meta-analyses on effects sizes for pain according to study design, duration of treatment, quality of study, and type of pain condition. Results suggest that randomized controlled trials with SMD −0.82 (95% CI, −1.20 to 0.53) and higher quality evidence SMD −0.88 (95% CI, 1.55 to −0.21) have marginally better pain outcomes than overall effects at −0.74 (95% CI, −0.97 to −0.52), while treatment duration appears to be similar to these overall effects in short, medium, and long interventions. Authors suggest improvements are most consistent for back pain and rheumatoid arthritic conditions. The remaining reviews do not provide enough data to perform subgroup analyses for gender, age, setting, or physiological intensity of yoga intervention. 

## 4. Discussion

### 4.1. Summary of Main Results

The following 13 chronic health conditions in adult populations are included in this overview: anxiety, arthritis, asthma, carpal tunnel syndrome, diabetes, epilepsy, fibromyalgia, hypertension, kidney disease, metabolic syndrome, pain, psychological health in cancer patients, and psychiatric disorders. Acute health conditions are included for women in pregnancy, labor, and menopause. 

#### 4.1.1. Interventions and Outcomes

Systematic reviews list some components of yoga interventions: breathing exercises (pranayama), physical postures (asanas), meditation (dhyana) and some yoga philosophy including sahaja (spontaneous movement), yama (personal restraint), and niyama (observance of yoga) teachings. Inconsistent reporting of changes in effect sizes of yoga by intervention type, delivery mechanism, setting, frequency, or duration of sessions highlights a serious gap in the literature and serious limitation in the overview findings. Of 13 systematic reviews that report geographical location, all include data collected from patients in North America, five include participants from Asia, and three reviews include studies from Europe. Fifteen reviews did not provide information on the setting of the intervention. Nine systematic reviews included delivery in a clinic or hospital setting, while two include a home-based intervention and one community-based intervention. 

As yoga research remains in the early stages of development, researchers appear to be more concentrated on outcome effects with clinical endpoints. However, traditional yoga practitioners claim that positive influence occurs in several health-related areas such as eliminating alcohol use, encouraging vegetarian diets, and providing an opportunity to increase social cohesion and positive group effects. These outcomes could relate more to mediating effects of yoga and warrant further investigation.

#### 4.1.2. Unclear Effects of Yoga—15 Systematic Reviews

The following outcomes were associated with unclear effects following yoga intervention: anxiety [18, 19], arthritis [14, 23], asthma [29], body mass index [9], diabetes management [8, 16], muscular strength [15], epilepsy [30], hypertension [12], and in pain for the elderly population [25]. Conclusions for menopause and carpal tunnel syndromes were split between positive and unclear effects. The more recent reviews in both instances show positive effects.

#### 4.1.3. Positive Effects of Yoga—11 Systematic Reviews

Seven of the systematic reviews assess pain management as a primary outcome. Of these reviews, 5 authors conclude positive effects of yoga [10, 20, 28, 31, 33]. Positive results for the treatment fibromyalgia are noted in one systematic review [20]. Potential improvements for anxiety and quality of life in cancer patients are noted in two reviews [22, 33]. One systematic review in psychiatric disorders concludes that yoga may be an effective and far less toxic adjunct treatment option for severe mental illness to prevent weight gain and patients' risk for cardiovascular disease [11].

#### 4.1.4. Adverse Effects of Yoga—No Systematic Reviews

Systematic reviews universally report that yoga is safe and no adverse effects of yoga treatment are reported. As yoga therapy in the reviews was usually instructor-led in a clinical setting, yoga delivered without a trained instructor may increase risk of injury and other adverse events.

#### 4.1.5. Size of Effect

**Figure 2:**
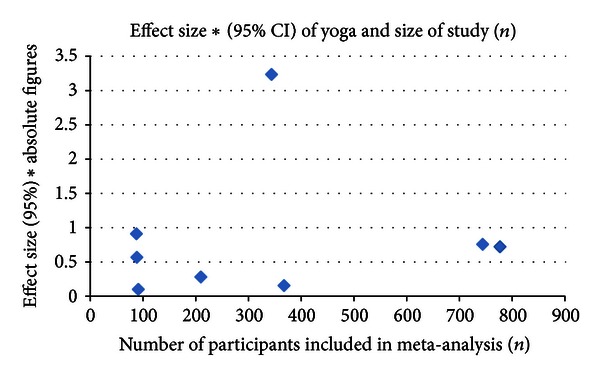
Effect size of yoga in comparison to study size.

In pooled analyses, statistical data report positive effects in five of six primary health outcomes for pain and various psychiatric disorders (depression, anxiety, PTSD, and schizophrenia). Effect sizes range from SMD −0.54 (95%  CI, − 0.96  to − 0.11; *P* = 0.01) for pain in fibromyalgia patients (VAS) and SMD −3.25 (95%  CI, − 5.36  to − 1.14; *P* = 0.002) in various psychiatric disorders (BDI, HADS, etc.). In the first instance, water yoga and awareness of yoga versus waitlist and treatment shows benefit. Ten studies using integrated yoga, Sudarshan, Kriya, Hatha, and Iyengar techniques favor yoga over other treatments and control groups, although the details are not reported. Most of the systematic reviews cite methodological weaknesses for unclear results, attributing this to small sample sizes and limited numbers of high-quality studies available for review. To investigate the impact of study size and quality on yoga's effect size on health outcomes, see Figure 2. Although limited by six quantitative data points, it does not appear that study size correlates with yoga's size of effect. 

### 4.2. Limitations of This Review

#### 4.2.1. Data Characteristics

The quality and quantity of evidence is a limitation to this overview. Though the quality of systematic reviews is high (9.4 AMSTAR), the quality of evidence included in reviews is generally low (GRADE). Important variables such as population statistics including gender, age, duration of interventions, comorbidities, and socioeconomic status are often not reported, limiting the potential for subgroup and meta-analyses. Of the primary and secondary outcome measures reviewed, no reports for all-cause mortality, hospital referral rates, cost effectiveness, or psychosocial behavioral changes are included which suggests at least four areas of potential investigation. 

In two reviews that assess publication bias, one funnel plot that includes pain outcomes [10] did not reveal any significant symmetry, while the other review for psychiatric disorders indicates an asymmetric plot and publication bias [11]. The remaining 24 reviews do not provide results of Egger's regression, funnel plot, or critical analysis of publication bias; therefore, the degree to which positive outcomes are influenced by publication bias is not known.

As all reports are written in English and the majority of reviews found on electronic databases include studies from the Western hemisphere, it is possible that existing reviews have been missed. The transferability of results may be limited due to only partial descriptions of interventions such as asana, pranayama, and meditative techniques. A broader definition of “systematic review” might increase the number of reviews included from diverse backgrounds, though strict criteria in terms of systematic review quality limits the inclusion of low-quality reports. Missing data for follow-up measures, characteristics of yoga intervention, and components of yoga therapy limit the confidence and number of conclusions that can be drawn, though this lack of data may be due to weakness in sources from primary studies and not necessarily a flaw in systematic review methodology. 

#### 4.2.2. Sources of Heterogeneity

Review authors identify types of yoga intervention, population characteristics, outcome measures, and study designs as sources of heterogeneity. As a result of this heterogeneity, most reviews consider independent studies in their analyses. Results are pooled in only six instances, where statistical heterogeneity was found in three cases and one did not report. As a complex intervention, some heterogeneity is inevitable with yoga and in fact desirable to replicate real-life circumstances. Study designs could be improved to focus on specific interventions.

#### 4.2.3. Duplication of Primary Studies

Duplication of primary studies appears in 40 cases across 17 reviews (yoga-only reviews: [8, 11, 14, 18, 21, 27, 28, 53]; multiple interventions: [13, 16, 17, 23–26, 31, 33]). The highest incidence of primary study overlap occurs in pain [25, 53] and menopause reviews [17, 21]. In further analysis, when the Garfinkel studies are removed, two systematic reviews are eliminated from this review [23, 26]. For pain, the more recent Bussing study concludes positive effects with yoga intervention, while Morone concludes unclear effects using similar studies. The removal of these two studies from the pool of results does not appear to change the net positive effects of yoga for pain conditions. In menopause, although 4 of 7 articles in each review are duplicates, authors' conclude different results: Lee et al. [21] suggest unclear effects of yoga, while Innes et al. [17] suggest positive effects of yoga on menopausal symptoms.

#### 4.2.4. Date of Search

The rate of publication for yoga systematic reviews is increasing rapidly. In an updated search (March 1, 2013), nine of 17 new titles pass initial screening for inclusion. Screening of abstracts identifies seven of these reviews that would need to be collected for further inclusion analysis, of which three focus on adult cancer [54–56], one on chronic obstructive pulmonary disease [57], one for depression [58], one for anxiety [59], and one for phantom limb pain [60]. These reviews suggest positive impact of yoga for primary outcomes with no adverse effects, though authors unanimously state that more and better-quality research is needed. In a recent overview of yoga, authors conclude there is relatively high-quality evidence to suggest that yoga may have beneficial effects for pain-associated disability and mental health [53], conclusions that are further substantiated by this overview.

## 5. Conclusion

### 5.1. Implications for Practice

Yoga for treatment of acute and chronic health conditions is not likely to exacerbate symptoms in an experimental setting, although clear effect sizes and probabilities for beneficial outcomes in a specified population are not available at this time. Cumulative findings indicate that Hatha and Restorative yoga have the highest correlation with positive outcomes for managing pain symptoms, anxiety, and depression. Home study and instructor-led yoga (practiced 60 minutes 3 times per week) appear to have similar positive impacts. 

### 5.2. Implications for Research

This overview adds a comprehensive and methodical examination of yoga interventions in adult populations for treatment of acute and chronic health conditions. The findings do support earlier claims that depression, pain, and anxiety could be positively affected by yoga intervention, though evidence is positive but less significant in populations with cardiovascular risk factors, fibromyalgia, or autoimmune disease. It is evident that systematic reviewers and primary research teams should include more information with regards to the characteristics of yoga intervention, including type, frequency, duration, and physiological intensity of practice. Video-led yoga needs to be explored further as one review includes this delivery mechanism and yields positive results, though the sample size is small and adverse effects are not measured. Health outcomes in other adult populations for asthma, arthritis, carpal tunnel syndrome, epilepsy, diabetes, kidney disease, and menopausal women remain uncertain. Two earlier reviews (before June 1, 2012) and three newer systematic reviews investigate yoga's effect for adult cancer. These papers should inform future investigations in terms of patient-relevant outcomes such as pain management, immunological responses, anxiety, and health-related quality of life.

Yoga is a complex intervention that includes physical movement, breathing techniques, meditation, visualization and philosophical underpinnings that may influence attitudes, beliefs and social interaction. A new hypothesis informed by results of this overview, together with an emerging trend of increased yoga research for cancer populations, suggest the complex and varied nature of yoga may better serve patients who experience a cluster of symptoms that include psychological distress, fatigue, pain and a compromised health-related quality of life. Further study into these effects should include analysis of adherence rates, outcomes in morbidity, mortality rates, disease progression markers, physical function and long-term follow-up. 

## Figures and Tables

**Box 1:**
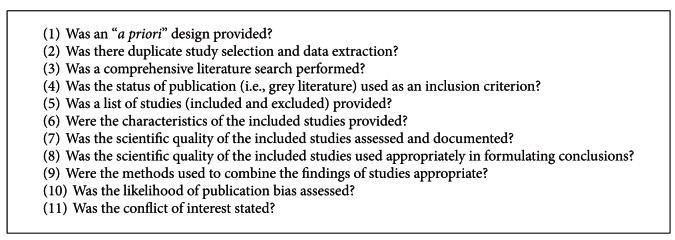
The AMSTAR tool criteria.

**Box 2:**
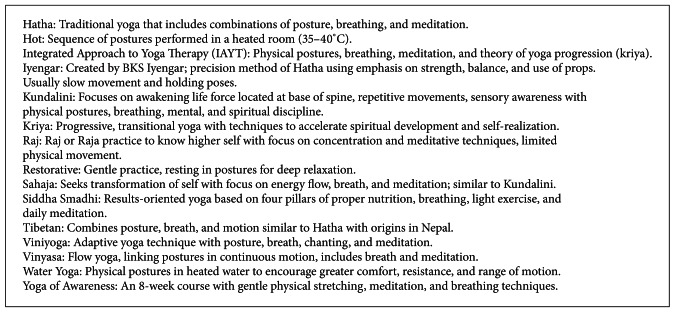
Types of yoga intervention.

**Box 3:**
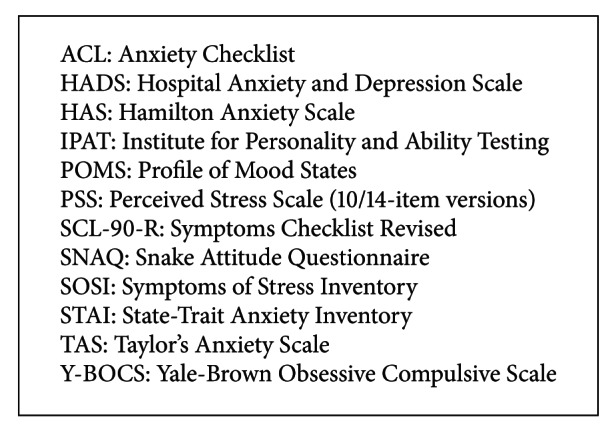
Summary of anxiety outcome measures.

**Box 4:**
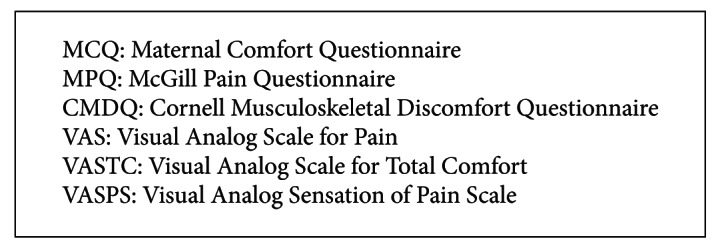
Summary of outcome measures for pain.

**Table 1:** Characteristics of included systematic reviews.

Review	Population			Type of yoga interventions	Type of comparisons	Outcomes
	Health condition (ICPC-2 class)	No. of primary studies incl. in SR	No. of participants			
*Aljasir et al., 2010 *[8]	Type 2 diabetes (T90)	5	362	Mixed yoga and yoga with dietary and herbal intake	No data	Blood glucose levels, lipid profile, body mass index (BMI), and diabetes-related complication

Anderson and Taylor, 2011 [9]	Metabolic syndromes (T99)	2	125	Restorative yoga	Waitlist, usual care	Body mass index (BMI), waist circumference, blood pressure, insulin sensitivity, blood glucose, blood lipids (HDL, LDL), perceived stress (SF-36), and food frequency

*Büssing et al., 2012 * [10]	Pain symptoms (A1; N1; L)	16	937	Mixed yoga (LAYT, Viniyoga, Raj, and unspecified others)	Waitlist, usual care, exercise, medication, and lecture	Effect size of pain using standardized measurements (i.e., VAS, MPQ, and CMDQ)

*Cabral et al., 2011* [11]	Psychiatric disorders (P99)	10	343	Hatha, Iyengar, Kriya, Sahaja, Integrated, Meditation, and Siddha Samadhi	No data	Major psychiatric disorders (schizophrenia, depression, anxiety, and PTSD)

Dickinson et al., 2008 [12]	Hypertensive (K86)	2	63	Yoga and meditation	No comparison group	Systolic and diastolic blood pressure

Gerritsen et al., 2002 [13]	Carpal tunnel syndrome (N93)	1	42	11 yoga postures	Usual care	Pain and sleep disturbance scores

*Haaz and Bartlett, 2011* [14]	Arthritis (L91)	10	243	Iyngar and Kundalini yoga	Waitlist, usual care, and no comparison group	Disease activity score (DAS), joint inflammation, functional ability (strength, balance, flexibility, and mobility), psychosocial (arthritis impact measurement scale 2 (AIMS2), HRQL (SF-36), Beck Depression Index (BDI)), and medication usage

Heiwe and Jacobson, 2011 [15]	Chronic kidney disease (U14)	1	40	Modified yoga including balancing, strengthening, and breathing techniques	Exercise	Muscular strength, grip strength, and blood lipids (triglyceride, total cholesterol, and HDL cholesterol)

Innes and Vincent, 2007 [16]	Women with menopause (X11)	7	408	Hatha, Iyengar, Sahaja, Hot, Yoga Awareness Program (Kripalu), and Integrated Approach to Yoga Therapy	Waitlist, exercise, and no comparison group	Overall menopausal symptoms, vasomotor menopausal symptoms, and sleep disturbance

Innes et al., 2010 [17]	Type 2 diabetes (T90)	25	1828	Various components including asana, Kriya, or cleansing exercises, meditation, pranayama, and meditation	Waitlist, usual care, exercise, and book/pamphlet	Insulin resistance (fasting glucose, postprandial glucose, fructosamine, fasting insulin, and fasting glycated hemoglobin), blood lipid profile (cholesterol, triglycerides, low/high-density lipoprotein, very LDL, cholesterol/HDL ratio, and LDL/HDL ratio), anthropometric measures (BMI, body weight, and body composition), blood pressure (systolic/diastolic), and medical usage

*Kirkwood et al., 2005* [18]	Anxiety disorders (P74)	8	370	Kundalini	Sham yoga, medication, book/pamphlet, and tablet placebo	Anxiety scales and checklists (YBOCS, HAS, ACL, Inst. for personality and ability testing)

*Krisanaprakornkit et al., 2006* [19]	Anxiety disorders (P74)	2	76	Kundalini	Relaxation response/mindful meditation	Anxiety rating scale

Langhorst et al., 2012 [20]	Women with fibromyalgia syndrome (A99)	2	93	Yoga of Awareness Program	Waitlist, usual care	Pain, sleep, fatigue, depression (VAS), and health-related quality of life (FIQ—fibromyalgia impact questionnaire)

*Lee et al., 2009* [21]	Menopausal women (X2)	7	470	Iyengar, restorative, and integrated yoga (!AYT)	Waitlist, exercise	Psychological, somatic, vasomotor, and total symptoms of menopause

*Lin et al., 2011* [22]	Psychological Health (P1; P3; P29)	10	788	Hatha, restorative, integrated, mind-body stress reduction, and Tibetan yoga	No data	Psychological health rating (anxiety, depression, distress, and stress), and quality of life and physical measures (self-reported health, fatigue)

Mahendira and Towheed, 2009 [23]	Osteoarthritis (L91)	1	No data	Unspecified yoga	Waitlist with wrist splint	Efficacy of treatment

Marc et al., 2011 [24]	Anxiety in pregnant women (P73; W78)	1	34	Mindfulness interventions	Waitlist	Perceived stress (perceived stress scale), depression, anxiety, positive/negative affect, and affect regulation

Morone and Greco, 2007 [25]	Pain symptoms (A1)	4	188	Yoga, relaxation, and education	Waitlist, book/pamphlet	Pain (WOMAC, RDS, joint tenderness, and visual analogue scale), physical function

Muller et al., 2004 [26]	Carpal tunnel syndrome (N93)	1	46	Unspecified yoga	Usual care (splint)	Pain and grip strength

*Pilkington et al., 2005* [27]	Depression (P76)	5	183	Iyengar, Kriya, and Broota's relaxation technique (BRT)	Waitlist, sham yoga, exercise, medication, and electroconvulsive therapy	Depression rating scale

*Posadzki et al.,* *2011* [28]	Pain symptoms (A1)	7	403	Hatha, Iyengar, Viniyoga, and 2 others unspecified	Waitlist, usual care, exercise, book/pamphlet	Pain, disability, depression rating scales, and medication usage

Ram et al., 2003 [29]	Asthmatic (R96)	3	147	Breathing exercise, meditation, postures, deep muscle relaxation, and chanting	Waitlist, exercise, and no data for some groups	Asthma symptoms (peak expiratory flow rates, exacerbations per week, and asthma symptom scores)

*Ramaratnam and Sridharan, * *2000* [30]	Epileptic (N88)	2	50	Sahaja, Pranayama, Dhyana, Yama, and Niyama	Sham yoga, talk therapy	Seizure frequency and duration

Slade and Keating, 2007 [31]	Low back pain (L33)	2	145	Iyengar, Viniyoga	Exercise, lecture, and book/pamphlet	Pain and function rating scores

Smith and Pukall, 2009 [32]	Pain/relaxation in labour (P01; W78)	2	281	Yoga program with educational activities	Usual care, exercise, music, and other yoga groups	Pain intensity, satisfaction with pain relief, satisfaction with childbirth, Apgar score, use of pharmacological pain relief, length of active labour, and augmentation in labour

*Smith et al., 2011* [33]	Anxiety in cancer patients (P29; A79)	10	892	Hatha, Iyengar, Restorative, Yoga of Awareness, Tibetan, and 4 others unspecified	Waitlist, exercise, and talk therapy	Anxiety, stress, depression, fatigue, sleep quality, spiritual wellbeing, and quality of life scales

Italics: systematic reviews including only yoga interventions.

Normal: systematic reviews including yoga interventions plus other interventions.

**Table 2:** Characteristics of excluded reviews (ordered by review author).

Review (author, year)	Reason for exclusion
Alexander et al., 2008 [34]	This study did not satisfy Oxman criteria of a systematic review
Beddoe and Lee, 2008 [35]	This study did not satisfy Oxman criteria of a systematic review
Brotto et al., 2009 [36]	This study did not satisfy Oxman criteria of a systematic review
Burgess et al., 2011 [37]	This study population includes children
Innes et al., 2005 [38]	This study population includes children and healthy adults
Kozasa et al., 2010 [39]	This study did not satisfy Oxman criteria of a systematic review
Krisanaprakornkit et al., 2010 [40]	This study population includes children
Lynton et al., 2007 [41]	This study does not include a randomised control or controlled trial of yoga
Mehta and Sharma, 2010 [42]	This study did not satisfy Oxman criteria of a systematic review
Posadzki et al., 2011 [28]	This study population includes children
Posadzki and Ernst, 2011 [43]	This study population includes children
Shen and Nahas, 2009 [44]	This study did not satisfy requirements of Oxman criteria of systematic review; no yoga interventions in a RCT/CT
Steurer-Stey et al., 2002 [45]	This study did not satisfy Oxman criteria of a systematic review
Towheed, 2005 [46]	This study did not satisfy Oxman criteria of a systematic review
Vickers and Smith, 1997 [47]	This study population includes children

**Table 3:** Overview of reviews: quality and outcomes summary.

Review (first author, year)	Primary outcome (as stated by review author or first listed)	Quality rating of SRs (AMSTAR)	Quality rating of evidence (grade)					SR authors' conclusions		
			Very low	Low	Moderate	High	Insufficient data to assess	Positive effect	Negative effect	Unclear effect
Aljasir, 2010 [8]	Management of type II diabetes	10		*◆*						*◆*
Anderson, 2011 [9]	Body mass index	9.5					*◆*			*◆*
Büssing, 2012 [10]	Pain (effect size)	11		*◆*				*◆*		
Cabral, 2011 [11]	Treatment for psychiatric disorder	11					*◆*	*◆*		
Dickinson, 2008 [12]	Blood pressure	10.5	*◆*							*◆*
Gerritsen, 2002 [13]	Pain (carpal tunnel syndrome)	8.5		*◆*						*◆*
Haaz, 2011 [14]	Clinical outcomes in arthritis	6	*◆*							*◆*
Heiwe, 2011 [15]	Muscular strength	11			*◆*					*◆*
Innes, 2007 [16]	Metabolic and anthropometric measures for diabetes mellitus	9					*◆*	*◆*		
Innes, 2010 [17]	Menopausal symptoms	6.5					*◆*	*◆*		
Kirkwood, 2005 [18]	Treatment for anxiety	10		*◆*						*◆*
Krisanaprakornkit, 2006 [19]	Treatment for clinical anxiety	10.5	*◆*							*◆*
Langhorst, 2012 [20]	Pain (fibromyalgia)	10.5		*◆*				*◆*		
Lee, 2009 [21]	Menopausal symptoms	11		*◆*						*◆*
Lin, 2011 [22]	Quality of life for cancer patients	10		*◆*				*◆*		
Mahendira, 2009 [23]	Effectiveness of treatment for osteoarthritis	8.5					*◆*			*◆*
Marc, 2011 [24]	Perceived stress	11		*◆*						*◆*
Morone, 2007 [25]	Pain (chronic in older adults)	9.5					*◆*			*◆*
Muller, 2004 [26]	Effectiveness of treatment for CTS	10.5		*◆*				*◆*		
Pilkington, 2005 [27]	Treatment for depression	7					*◆*			*◆*
Posadzki, 2011 [28]	Pain (low back)	10		*◆*				*◆*		
Ram, 2003 [29]	Asthma symptoms	6					*◆*			*◆*
Ramaratnam, 2000 [30]	Treatment for epilepsy	11		*◆*						*◆*
Slade, 2007 [31]	Pain (low back)	5.5					*◆*	*◆*		
Smith, 2009 [32]	Psychological functioning of patients with cancer diagnosis	10.5		*◆*				*◆*		
Smith, 2011 [33]	Pain (labour)	9		*◆*				*◆*		

Total (average)		9.4	3	13	1	0	9	11	0	15

**Table 4:** Overview of reviews—primary outcomes (yoga meta-analyses).

Review	Condition	Outcome		Intervention(s)	Comparison	Effect size (95% CI)	*P* value	No. of participants (studies)	Quality of Evidence (grade)	Heterogeneity analysis	Comments
		Description	Measuring instrument(s)								
Büssing et al., 2012 [10]	Pain	Effect size of pain	VAS, MPQ, CMDQ	Hatha, Iyengar, and unspecified yoga	Physical activity, educational sessions, waiting list, lecture, routine care, and conversation	SMD −0.74 (−0.97, −0.52)	<0.0001	776 (12)	Moderate* (low)	Chi square test 19.73, df = 11 (*P* = 0.05); *I*-square = 44% Moderate heterogeneity detected	“Methodological quality of the studies had no relevant impact on the study outcome; of note, studies with higher quality had a better pain outcome as compared with studies with low quality”

Cabral et al., 2011 [11]	Psychiatric disorder	Treatment of psychiatric disorder: depression, anxiety, PTSD, and schizophrenia)	BDI, HADS, digit span test, wellbeing scores, stress hormone levels (cortisol and ACTH)	Integrated, Sudarshan Kriya, Hatha, Sahaj, Meditation yoga, Siddha Samadhi, and Iyengar	Other treatment	SMD −3.25 (−5.36, −1.14)	0.002	343 (10)	n.r	Cochran *Q* test = 369.69 (*P* < .001) for fixed model data Heterogeneity indicated	“Funnel plot and Egger regression test (*P* = 0.007) indicate publication bias; failsafe *N* = 212 interpreted to suggest 21.2 missing studies needed to nullify observed effect”

Langhorst et al., 2012 [20]	Fibromyalgia syndrome	Pain	VAS	Yoga of Awareness, water yoga	Waitlist, treatment as usual	SMD −0.54 (−0.96, −0.11)	0.01	88 (2)	(Low)	*I*-square = 0%	“Evidence of a short-term relief of four key domains of FMS by Yoga: pain, fatigue, depression, quality of life” No evidence of sleep improvement with yoga v. active or waitlist controls

Lee et al., 2009 [21]	Menopause	Menopausal symptoms	MENSI	Iyengar, unspecified yoga	No treatment	SMD 0.07 (−0.25, 0.39)	0.66	91 (2)	2.5/5* (low)	Chi square test 0.28, df = 1 (*P* = 0.60); *I*-square = 0% No heterogeneity detected	“Evidence is insufficient to suggest that yoga is an effective intervention for menopause”

Lin et al., 2011 [22]	Psychological health, quality of life, and physical health of cancer patients	Anxiety, depression, distress, and stress	HADS, PSS, STAI, SOSI, POMS, SCL-90-R, STAI, CES-D, PANAS, IES, DMI	Integrated yoga, MBSR: gentle yoga, Tibetan, and unspecified yoga	Waitlist, n.r	SMD −0.95 (−1.63, −0.27)	0.006	744 (10)	5.25/10** (low)	Chi square test 33, 96, df = 4 (*P* = 0.006); *I*-square = 88% Very high heterogeneity detected	“Findings show potential benefits of yoga for people with cancer in improvements of psychological health…clinical heterogeneity to be considered when interpreting results”
		Quality of life	SF-12, FACT_B, FACT_G, and EORTC QLQ-C30	Restorative, Hatha, and unspecified yoga	n.r	SMD −0.29 (−0.58, 0.01)	0.51	210 (3)	4.7/10** (low)	Chi square test 1.34, df = 2 (*P* = 0.60); *I*-square = 0% No heterogeneity detected	
		Physical health	SF-12, FACT_B, and FACT_G	Restorative, Hatha, Tibetan, and MBSR: gentle yoga	n.r	SMD −0.16 (−0.37, −0.06)	0.15	367 (4)	5.25/10** (low)	Chi square test 3.96, df = 3 (*P* = 0.15); *I*-square = 24% Some heterogeneity detected	

Slade and Keating, 2007 [31]	Chronic low back pain	Medium-term pain	n.r	Viniyoga, Iyengar	Trunk strength and aerobics, book and lectures	SMD 0.92 (0.47, 1.37)	n.r	88 (2)	7.7/10** (n.r)	n.r	“Indicated significant and large effects for medium-term pain in favour of yoga”

n.r: not reported; BDI: Beck Depression Inventory; VAS: Visual Analogue Scale; MENSI: Menopausal Self-inventory; MPQ: McGill pain questionnaire; PPI: Present Pain Index; CMDQ: Cornell Musculoskeletal Discomfort Questionnaire; HADS: Hospital Anxiety and Depression Scale; PSS: Perceived Stress Scale; STAI: State of Trait Anxiety Inventory; SOSI: Symptoms of stress inventory; POMS: Profile of Mood States; SCL-90-R: Symptoms Checklist Revised; CES-D: Center for Epidemiologic Studies Depression Scale; PANAS: Positive and Negative Affect Schedule; IES: Impact of Events Scale; DMI: Distressed Mood Index; SF-36: Medical Outcomes Study Short-Form Health Survey; SF-12: The 12-Item Short Form Health Survey; FACT_B: Functional Assessment of Cancer Therapy-Breast; FACT_G: Functional Assessment of Cancer Therapy-General; EORTC QLQ-C30: European Organization for research and Treatment of Cancer Quality of Life Questionnaire Version 3.0; MBSR: Mindfulness-based stress reduction.

*Average Jadad score.

**Average PEDro scale.

## Appendix

### Electronic Search Protocol

Identification of Relevant Databases:

1. Cochrane Library
2. CENTRAL
3. MEDLINE
4. EMBASE
5. AMED
6. PsycINFO
7. CINAHL
8. IndMED
9. CAMQuest
10. Scopus.

The Electronic Search Performed in May 2012

1. Online access via SOLO [http://solo.bodleian.ox.ac.uk with SSO password]
2. Enter free text terms, MeSH descriptors and set filters
3. Scan results for relevant titles
4. Scan titles for relevant abstracts
5. Scan abstract for relevant review articles
6. Save citations with abstracts to a file and transfer to reference management database [sente]
7. Collect relevant articles in.pdf and save to file on external and internal computer hard drives under review identification label
8. Store the external hard drive in separate location under lock and key. Two key holders.

Cochrane Database of Systematic Reviews (via Cochrane Library, Wiley)

1. yoga
2. yogi*
3. asana
4. pranayama
5. dhyana
6. meditation
7. 1 or 2 or 3 or 4 or 5 or 6.

MEDLINE (1946-), EMBASE (1974-), AMED (1985-), PsycINFO (1960-) (via OVID)

1. MeSH descriptor; Meditation; Relaxation Therapy; Mind Body Medicine explode all trees
2. (yoga OR yogi* OR asana OR pranayama OR dhyana OR meditation)
3. MeSH descriptor; Meta-analysis; Review explode all trees
4. (systematic OR review OR meta-analysis)
5. 1 OR 2
6. 3 OR 4
7. 5 AND 6.

CINAHL (via EBSCOHost)

1. limit: publication type (meta-analysis); exclude (MEDLINE results)
2. (yoga OR yogi* OR asana OR pranayama OR dhyana OR meditation)
3. (systematic OR review OR meta-analysis)
4. 1 AND 2.

IndMED (http://indmed.nic.in); CAMQuest (http://www.cam-quest.org/en/)

1. (yoga OR yogi* OR asana OR pranayama OR dhyana OR meditation)
2. (systematic OR review OR meta-analysis)
3. 1 AND 2

Scopus (via SciVerse; Elsevier)

1. limit: publication type (review)
2. (yoga OR yogi* OR asana OR pranayama OR dhyana OR meditation)
3. (systematic OR review OR meta-analysis)
4. 2 AND 3.
